# A Malaria Vaccine Based on the Polymorphic Block 2 Region of MSP-1 that Elicits a Broad Serotype-Spanning Immune Response

**DOI:** 10.1371/journal.pone.0026616

**Published:** 2011-10-26

**Authors:** Graeme J. M. Cowan, Alison M. Creasey, Kelwalin Dhanasarnsombut, Alan W. Thomas, Edmond J. Remarque, David R. Cavanagh

**Affiliations:** 1 Institute of Immunology and Infection Research, University of Edinburgh, Edinburgh, United Kingdom; 2 Biomedical Primate Research Center, Rijswijk, The Netherlands; State University of Campinas, Brazil

## Abstract

Polymorphic parasite antigens are known targets of protective immunity to malaria, but this antigenic variation poses challenges to vaccine development. A synthetic MSP-1 Block 2 construct, based on all polymorphic variants found in natural *Plasmodium falciparum* isolates has been designed, combined with the relatively conserved Block 1 sequence of MSP-1 and expressed in *E.coli*. The MSP-1 Hybrid antigen has been produced with high yield by fed-batch fermentation and purified without the aid of affinity tags resulting in a pure and extremely thermostable antigen preparation. MSP-1 hybrid is immunogenic in experimental animals using adjuvants suitable for human use, eliciting antibodies against epitopes from all three Block 2 serotypes. Human serum antibodies from Africans naturally exposed to malaria reacted to the MSP-1 hybrid as strongly as, or better than the same serum reactivities to individual MSP-1 Block 2 antigens, and these antibody responses showed clear associations with reduced incidence of malaria episodes. The MSP-1 hybrid is designed to induce a protective antibody response to the highly polymorphic Block 2 region of MSP-1, enhancing the repertoire of MSP-1 Block 2 antibody responses found among immune and semi-immune individuals in malaria endemic areas. The target population for such a vaccine is young children and vulnerable adults, to accelerate the acquisition of a full range of malaria protective antibodies against this polymorphic parasite antigen.

## Introduction

Antibody responses to polymorphic parasite antigens of *Plasmodium falciparum* are associated with protective immunity to malaria [1], [2], [3], [4], [5]. There is evidence that selective pressure by the human immune system may be responsible for the evolution and maintenance of such polymorphism [6], . Protective natural immunity to malaria develops only after repeated infection [9], [10], suggesting that exposure to different variants of polymorphic antigens may be required to develop a repertoire of variant antibodies before adequate protection can be achieved [11]. The development of vaccines against protective but polymorphic antigens would accelerate the acquisition of a broad immune repertoire, particularly in infants, young children and other specific vulnerable groups. It seems likely that selective immune pressure on antigens which elicit antibodies most threatening to the parasite's survival has driven the evolution and maintenance of this polymorphism [8].

Merozoite surface protein 1 (MSP-1) is the most abundant surface component of the merozoite stage of the parasite life cycle, making up 40% of the GPI-anchored merozoite surface protein coat [12], [13], [14]. MSP-1 is accessible to the host immune system, since it remains on the surface of the merozoite while it is free of the host erythrocyte [15]. Monoclonal antibodies raised against the MSP-1 molecule specifically recognize all forms of the erythrocyte stages of the parasite [16], [17], [18], and MSP-1 is likely to be a target of cytotoxic T cell responses due to its expression in hepatic liver schizonts [19], [20]. An N-terminal region of MSP-1, known as Block 2, is by far the most polymorphic region of the molecule, with hundreds of known variant sequences from globally diverse parasite isolates [21], [22]. Several sero-epidemiological studies have shown that antibodies to Block 2 are associated with reduced risk of clinical malaria episodes [3], [7], [23], [24]. Other parts of the MSP-1 molecule, such as MSP-1_19_ showed little or no such association with protection [25], [26]. The immune response to Block 2 is almost exclusively of the IgG3 subclass unlike the response directed to MSP1_19_, where the predominant subclass is IgG1 [27], [28]. *In vitro* ADCI assays with purified IgG3 from immune individuals (including antibodies to MSP-1 Block 2) have shown the importance of this subclass as an inhibitor of parasite growth [29], [30], supporting the hypothesis that antigens that elicit IgG3 responses (such as MSP-1 Block 2 and MSP-2) are important targets of protective mechanisms [31], [32].

In an *in vivo* non-human primate model, we have demonstrated that immunization of highly susceptible *Aotus lemurinus griseimembra* monkeys with a Block 2 GST fusion protein can elicit immune protection against parasite infection in two of four immunized animals using a human compatible adjuvant (Cavanagh *et al.*, manuscript in preparation). Significantly, parasite-reactive (i.e. IFA) anti-Block 2 antibody titers were the strongest predictor of protection in this study. Furthermore, the animals that controlled their parasitaemia recognised a larger number of peptide epitopes within Block 2 compared to the non-protected animals. Both these lines of evidence suggest that the Block 2 region of MSP-1 is a target of protective immunity against *P. falciparum* and thus a promising candidate for the development of a malaria vaccine antigen.

**Figure 1:**
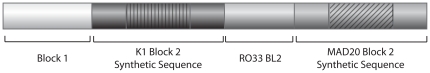
Schematic representation of the MSP-1 hybrid vaccine construct, based on the polymorphic N-terminal region of MSP-1. The construct encodes the N-terminal MSP-1 Block 1 region, the K1 Block 2 synthetic sequence, the RO33 Block 2 sequence and the MAD20 Block 2 synthetic sequence of MSP-1 of *P. falciparum*. Synthetic Block 2 repeat sequences of the K1 and MAD20 serotypes are indicated by vertical and diagonal hatched markings respectively.

Sequence analysis of more than 100 variants of the MSP-1 Block 2 sequence in naturally occurring *P. falciparum* isolates, and epitope mapping of natural antibody response to Block 2 in humans showed that despite their extreme polymorphism, there are 3 basic serotypes of Block 2, named after representative clones from each serotype as the K1, MAD20 and RO33 types. Within both the K1 and MAD20 serotypes there are semi-conserved flanking sequences, which enclose extremely polymorphic repetitive sequences [21], [22], [33]. These repeat sequences comprise tripeptide repeat patterns that are unique to each serotype. By contrast the RO33 serotype is largely conserved but has a limited number of point mutations [21], [22]. In this study a synthetic gene has been constructed comprising all the known polymorphic sequences for each of the three serotypes, in an arrangement similar to that of the naturally occurring Block 2 alleles, creating a construct longer than any known natural allele, but incorporating the majority of known antigenic and sequence diversity in Block 2 (Fig. 1). Combining multiple serotypes of such a polymorphic region of MSP-1 would therefore allow the induction of antibody responses to multiple Block 2 serotypes by administration of a single polypeptide, combining known human T cell and B cell epitopes.

In earlier work, we showed that MSP-1 Block 2 was immunogenic in mice when formulated with Alum, and fused to the carrier protein GST [33]. However, an individual Block 2 antigen (*P. falciparum* strain FVO Block 2) when expressed as a recombinant protein in *E.* coli, proved to be weakly immunogenic, using a variety of adjuvants (Fig. S1). This is probably due to the lack of T cell epitopes within these highly polymorphic Block 2 sequences, which are made up of hydrophilic, polar residues not commonly found within MHC binding motifs. However, there is strong published evidence from other groups that human and mouse T-cell epitopes exist in Block 1 of MSP-1, as well as in the junction between Block 1 and Block 2 [34], [35]. Quakyi et al [34] reported that one highly conserved peptide within MSP-1 Block 1 (VTHESYQELVKKLEALEDAV) was recognised by human T cell clones which secreted IFNγ and proliferated. Subsequently, Parra at al [35] showed that this T cell epitope and an adjacent epitope were able to induce MSP-1-specific cellular and humoral immune responses when injected into mice. Therefore, to improve immunogenicity by incorporating cognate T cell help, we added the Block 1 sequence to the synthetic Block 2 construct, thus reconstructing these two epitopes. This synthetic MSP-1 Block 1/Block 2 construct (referred to as MSP-1 hybrid) was designed without affinity tags and then codon-optimised for expression in *E. coli* (Fig. 1).

This MSP-1 hybrid antigen, together with a suitable adjuvant, is designed to produce a vaccine that will induce protective antibody responses to polymorphic MSP-1 Block 2 sequences, replicating the network of responses found among immune and semi-immune adults in malaria endemic areas. The target population for such a vaccine is therefore primarily young children and vulnerable adults who do not produce a broad range of protective antibodies to *P. falciparum*. Vaccines such as the MSP-1 hybrid may also be useful for boosting and broadening the immune responses in individuals who already make limited, serotype-specific responses to Block 2.

## Results

### Expression and purification of MSP-1 hybrid

**Figure 2:**
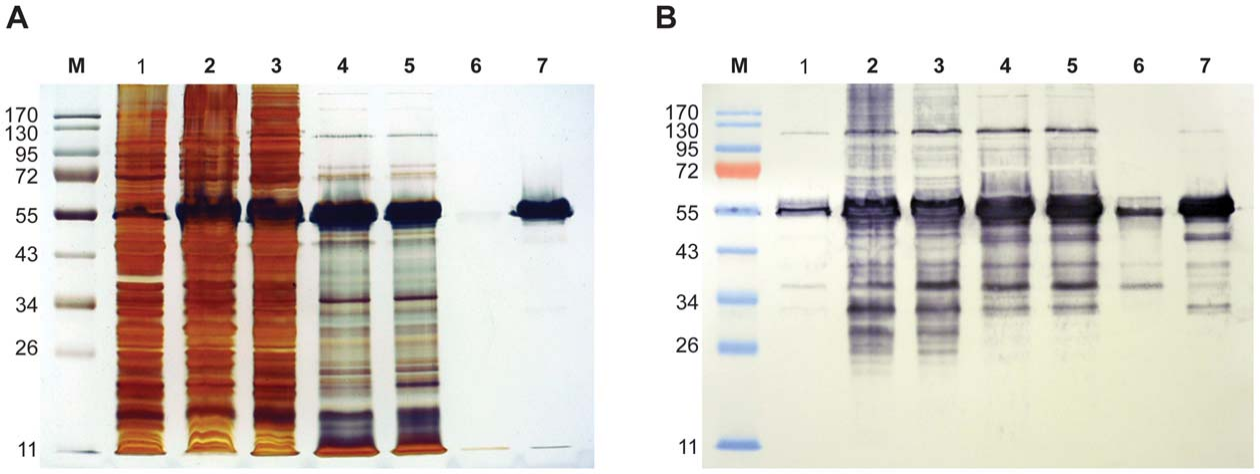
Analysis of protein expression and purification by gel electrophoresis and Western blotting. A. Samples from cell growth and purification steps were resolved by SDS-PAGE and silver stained. Cells harvested after induction of protein expression (lane 2) were lysed as described and subject to a two step purification process to obtain a final pure product. Lane 1, pre-induction cells; lane 2 post-induction cells; lane 3, soluble clarified cell lysate; lane 4 heat treated and clarified cell lysate; lane 5, dialysed pre-AEX sample; lane 6 Capto-Q flow through; lane 7, purified MSP-1 hybrid. B. Samples from cell growth and purification steps were resolved by SDS-PAGE and characterised by Western blotting. A nitrocellulose membrane blotted from an identically loaded gel to that shown in Panel A was probed with a monoclonal antibody specific for MSP-1 Block 2 (mAb 12.2, which recognises specific repeat sequences present in K1 serotype parasites). The mAb recognizes the monomeric form of the MSP-1 hybrid (∼55 kDa dominant band) and a dimeric form of the antigen (∼110 kDa band). Some degradation products are also detected by the mAb in all lanes, but these are not detectable by silver staining (Panel A).

The DNA sequence of the MSP-1 hybrid was codon optimized for expression in *E. coli*. The original coding sequence was sent to GeneArt AG, Regensburg, Germany, and optimized using the company's GeneOptimizer® software. The codon-optimized sequence contained no purification tag sequences, and consisted entirely of MSP-1-derived sequences, with the single amino acid addition of an N-terminal methionine initation codon. The synthesized gene was then cloned into the pET24a expression vector (Novagen, UK) and expressed in BLR(DE3)pLysS cells. Pilot expression tests were carried out to establish optimal small-scale expression conditions. Expression and induction conditions were modified, with cell growth at 30°C in Luria-Bertani medium containing 1% glucose and induction at 25°C with 1 mM IPTG for 4 h. This protocol resulted in high level expression of MSP-1 hybrid on induction as a soluble protein at ∼55 kDa in the cell lysate (Fig. 2A). The identity of the protein was confirmed by Western blotting using a mouse monoclonal specific for the K1 Block 2 serotype of MSP-1 (mAb12.2, Fig. 2B) [33] and with polyclonal sera raised against individual MSP-1 Block 2 recombinant proteins [33] (data not shown). The major soluble protein in the cell lysate was MSP-1 hybrid, with minor degradation products as detected by mAb 12.2 reactivity (Fig. 2B, Lane 2). In 1-liter shaking-flask culture, MSP-1 hybrid accounted for an estimated 15% of the total soluble protein, as assessed by densitometry of silver stained gels. The first purification step (70°C heating of cell lysate) eliminated the majority of host protein contaminants from the product, yielding a ∼80% pure product, equivalent to ∼210 mg L^−1^ cell culture. After dialysis against 25 mM Tris pH 8.0, and further purification by anion exchange chromatography, pure MSP-1 hybrid product (>97%) was obtained with a yield of 197.6 mg L^−1^ cell culture. When fractions from each purification step were probed by Western blotting with the anti-Block 2 mAb 12.2, some minor degradation species of the MSP-1 hybrid could be detected, but these were not visible on the equivalent silver-stained gel (Fig. 2A and 2B; lane 7).

This expression and purification process was easily transferred to 10-liter fermentor scale and to high density culture as described in Materials and Methods, with no loss of efficiency and/or protein yields (Table 1). During fermentor scale expression of the MSP-1 hybrid, there were no problems with plasmid stability or expression, despite the fact that the MSP-1 hybrid gene contains repetitive sequences, which have been reported to be problematic to express in *E. coli* [36]. Plasmid DNA from individual colonies, grown from harvested fermentor cell paste, contained identical MSP-1 hybrid sequences to the starter culture colonies (data not shown). The protein product does not appear to be toxic to cells, as fermentor cell growth continued after induction of protein expression with IPTG.

**Table 1:** Yield and purity estimates from fed-batch fermentor production of the MSP-1 hybrid.

	Whole Cell Pellet	Lysate	Post Heat Step	Final Product
**Total Protein (mg L^−1^)**	11034	9291	2051	550.6
**BB Hybrid Yield (mg L^−1^)**	2282	2520	1724	550.6
**Est. Product Purity %**	21	27	84	>97

Total protein yields were calculated by BCA assay on samples from each purification step. The specific MSP-1 hybrid content in each sample was determined by sandwich ELISA, using monoclonal 12.2 as a capture antibody and anti-MSP-1 hybrid polyclonal rabbit IgG as a detection reagent. MSP-1 hybrid content in samples from each purification step was calculated by interpolation from a standard curve, using serial dilutions of purified MSP-1 hybrid as capture antigen. Purity and yield were then calculated from these two measurements.

**Figure 3:**
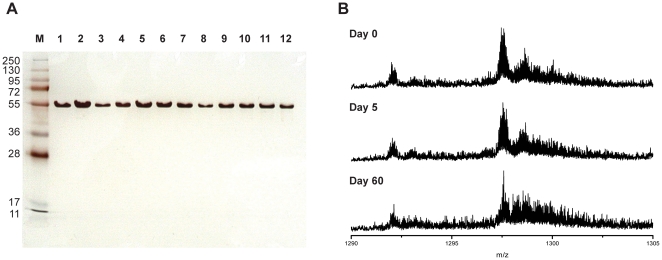
Stability analysis of purified MSP-1 hybrid protein. A. MSP-1 hybrid was incubated at 25°C for up to 60 days in liquid form in PBS, pH 7.2. Silver stained SDS-PAGE gels were used to estimate degradation of the MSP-1 hybrid over time. The final 60-day time point did not show any appreciable difference from the T_0_ sample. Lanes 1–12; samples taken at days 5,10,14,20,24,28, 30, 35, 42, 49, 56, and 60. B. MSP-1 hybrid samples incubated at 25°C (Panel A) were subject to mass spectrometric analysis as described in Materials and Methods. A single predominant species at *m/z* of ∼1297 was detected, which remained unaltered in mass over the 60 day incubation in liquid form. A minor species at *m/z* of ∼1292 is the MSP-1 hybrid minus its N-terminal methionine residue, a common modification which occurs to proteins expressed in *E. coli* [64].

Fermentor batch yields and purity of product were comparable with those obtained in shaking flask culture, with a final product containing no detectable contaminating proteins by silver staining of SDS-PAGE gels or by mass spectrometry (Fig. 2 and 3B). Highly pure (>97%) MSP-1 hybrid was recovered with a yield of 550 mg L^−1^ cell culture. Yields and purity of the purified product at each purification step from fed-batch fermentor studies are summarized in Table 1.

### Characteristics and stability of MSP-1 hybrid

The MSP-1 hybrid protein was found to have some atypical biochemical properties that are however characteristic of intrinsically unstructured proteins. MSP-1 hybrid migrated on standard reducing SDS-PAGE at ∼55 kDa, approximately twice both its predicted molecular mass (30,987 Da) and that determined experimentally by mass spectrometry (31,113 Da) (Fig. 2A). MSP-1 hybrid stained very weakly with Coomassie Brilliant Blue, but strongly with silver nitrate. The MSP-1 hybrid protein was observed to remain soluble in isotonic salt buffers at temperatures between 70°C–90°C. Remarkably, the purified MSP-1 hybrid antigen can be stored at ambient temperature without significant degradation. Purified MSP-1 hybrid was heat-treated at 70°C for 20 minutes, and then incubated at 25°C for 60 days. No significant breakdown of the protein was observed on SDS-PAGE gels (Fig. 3A). Sensitive analysis by mass spectrometry of samples taken before and after incubation at room temperature again showed only trace degradation, with some increased Mr species caused by salt adducts formed on prolonged incubation (Fig. 3B). Several monoclonal antibodies specific for individual serotypes of Block 2, (e.g mAb 12.2) and polyclonal mouse sera raised against individual Block 2 antigens, which recognise the native MSP-1 antigen in Western blots and IFA, were also shown to recognize the MSP-1 hybrid antigen in a specific and titratable manner (Fig. 2B and data not shown).

### Evaluation of immunogenicity and serotype specificity of the MSP-1 hybrid

**Figure 4:**
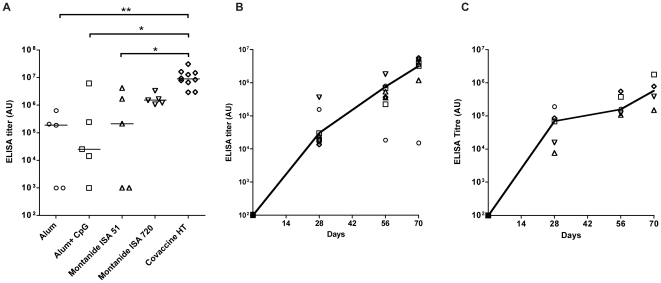
Immunogenicity and adjuvant testing of MSP-1 hybrid protein. A. Effect of adjuvants on antibody responses against MSP-1 hybrid. Groups of five or ten outbred MF1 mice were immunized s.c. three times at 4 week intervals with MSP-1 hybrid formulated with the adjuvants as indicated on the X axis. Two weeks after the last immunization, serum samples were tested by ELISA for antibody responses against MSP-1 hybrid. Titer was calculated as outlined in Materials and Methods. Data is shown on a log_10_ scale as dotplots of serum reactivity for individual animals with the median level of Ab reactivity for each group indicated by a horizontal line. Horizontal brackets indicate statistically significant differences between formulations as estimated by Kruskal-Wallis test. The asterisks represent statistical significance (*p<0.05, **p<0.01). B. Immunogenicity of the MSP-1 hybrid in rabbits. A group of eight rabbits were immunized i.m. three times at 4 week intervals with MSP-1 hybrid formulated in CoVaccine HT as described. At each immunization point, (d0, d28, d56) and two weeks after the last immunization (d70), serum samples from each animal were tested by ELISA for antibody responses against MSP-1 hybrid. Titer was calculated as outlined in Materials and Methods. Data is shown on a log_10_ scale as dotplots of serum reactivity for individual animals with the median level of Ab reactivity for each group indicated by the solid line. C. Immunogenicity of the MSP-1 hybrid in Rhesus monkeys. A group of five monkeys were immunized i.m. three times, at 4 week intervals with MSP-1 hybrid formulated in CoVaccine HT as described. At each immunization point, and two weeks after the last immunization (d70), serum samples from each animal were tested by ELISA for antibody responses against MSP-1 hybrid. Titers were calculated as outlined in Materials and Methods. Data is shown on a log_10_ scale as dotplots of serum reactivity for individual animals with the median level of Ab reactivity for each group indicated by the solid line.

Mice were immunized with MSP-1 hybrid in five different adjuvants suitable for use in humans, namely, aluminium hydroxide (Alhydrogel, +/− CpG 7909), Montanides ISA-51 and ISA-720 and CoVaccine HT. MSP-1 hybrid specific antibodies were detected by ELISA in the sera of the majority of immunized animals 14 days after the third dose, with median titers in each group ranging from 1.9×10^5^ (Alhydrogel) to 8.9×10^6^ (CoVaccine HT) (Fig. 4A). Montanide ISA-720 and CoVaccine HT gave the most consistent titers, with all animals producing significant titers of antibodies above 1.5×10^6^. Antibody titers were significantly higher in the CoVaccine HT group than all other groups, with the exception of the Montanide ISA-720 group (p<0.05 or less, Kruskal-Wallis test, Fig. 4A). In a single pairwise comparison, the CoVaccine HT group had significantly higher median titres than the Montanide ISA-720 group (8.9×10^6^ compared to 1.5×10^6^; Mann-Whitney U test, p = 0.0027, Fig. 4A).

**Figure 5:**
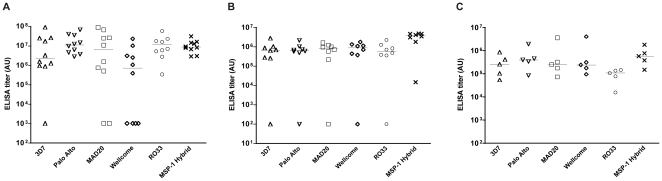
Serotype specificity of antibody responses to MSP-1 Block 2 in animals immunized with MSP-1. Serum samples at day 70 from groups of animals immunized with MSP-1 hybrid formulated with CoVaccine HT were tested by ELISA for reactivity with Block 2 GST proteins of different serotypes (K1 Block 2 serotype, 3D7 and Palo Alto; MAD20 serotype, MAD20 and Wellcome; RO33 serotype, RO33). ELISA titers were calculated as described in Materials and Methods. Data is shown on a log10 scale as dotplots of serum reactivity for individual animals with the median level of Ab reactivity for each group indicated by a horizontal line. A) Block 2 reactivity in immunized mice; B) Block 2 reactivity in immunized rabbits; C) Block 2 reactivity in immunized Rhesus monkeys.

Individual mouse sera from the CoVaccine HT immunisation group had broadly similar titers against individual K1-like type (3D7, Palo Alto), MAD20-like type (MAD20, Wellcome) and RO33 type antigens in most animals (Fig. 5A). However, some mice (but not rabbits or monkeys immunized with the CoVaccine HT formulation) selectively failed to respond to certain individual Block 2 antigens (Fig. 5A and 5B). Similar results were obtained for parasite-specific antibodies measured by immunofluorescence (IFA) against parasites of the same five serotypes (data not shown).

Based on the strong immunogenicity of MSP-1 hybrid in mice with five different adjuvant formulations, eight rabbits were then immunized with purified MSP-1 hybrid formulated with CoVaccine HT, using a similar schedule to that used in mice, but at 50 µg per dose (see Materials and Methods). Seven animals responded well to the immunisations, with one animal failing to respond to any significant extent (Fig. 4B). Broad serum reactivity against five recombinant MSP-1 Block 2 GST fusion proteins, representative of all three Block 2 serotypes, was observed in all 7 responding animals (Fig. 5B).

As part of a pre-clinical proof of principle experiment, a group of five Rhesus macaques (ranging 6.5 to 9.2 kg in body weight and 5.7 to 14.1 years of age at the time of first immunisations) were immunized three times with 0.5 mL containing 50 µg of the MSP-1 hybrid formulated in CoVaccine HT, using an identical protocol to that used in rabbits (above). In this study, the vaccine was delivered through the intramuscular route in alternating upper legs (left, right, left) at 0, 4 and 8 weeks [Fig. 4C]. The choice of the adjuvant for these studies was made on the basis that the adjuvant has already been used as an investigational adjuvant in clinical trials in humans (http://clinicaltrials.gov/ct2/show/NCT01015703) and that the MSP-1 hybrid vaccine formulated in CoVaccine HT was most effective in mice and rabbits (Figs. 4, 5 and above). The independent ethics committee at BPRC, constituted according to Dutch law on animal experiments, approved the study protocol (number DEC 598) prior to start of the experiment. Again, strong and consistent immunogenicity was elicited in all animals in response to the MSP-1 hybrid (Fig. 4C). Increasing titers were observed with each dose (median titers: day28, 6.9×10^4^; day56, 1.6×10^5^; day70, 5.8×10^5^). Encouragingly, antibody reactivity against individual MSP-1 Block 2 antigens, representative of the three Block 2 serotypes was also observed in all animals, indicating that the vaccine elicits a broad serotype antibody reactivity (median ELISA titers range from ∼1.0×10^5^ to ∼4.0×10^5^, Fig. 5C).

### Fine-scale epitope mapping of antibody responses to MSP-1 hybrid in immunized animals

**Figure 6:**
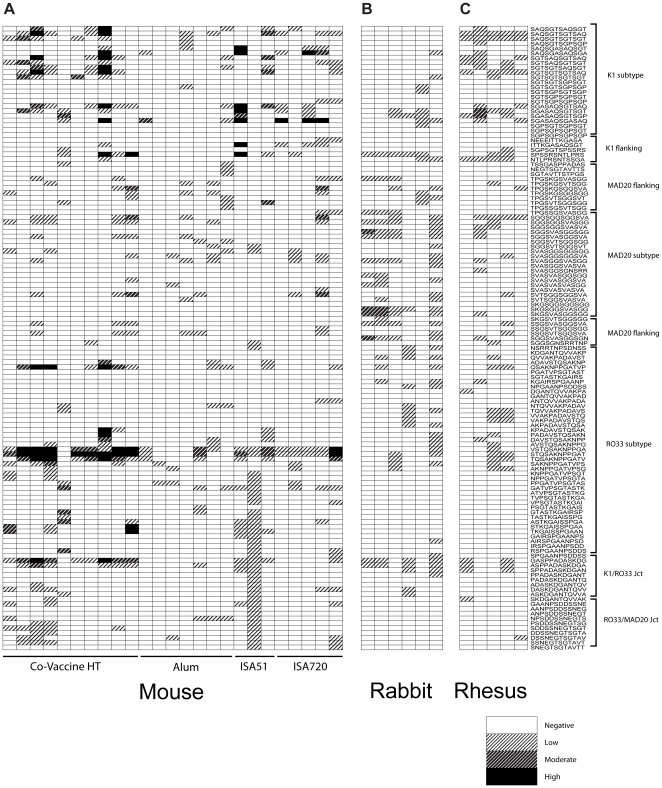
Recognition of peptide epitopes within the MSP-1 hybrid. A series of 133 N-terminally biotinylated dodecapeptides representing the sequence diversity of all three Block 2 serotypes were used in ELISA to map the antibody specificities present in the sera of immunized animals. Reactivity with individual peptides is shown as shaded boxes, with the depth of shading of each box representing the strength of reactivity of a 1∶1000 dilution of sera with each peptide. The sequences and Block 2 serotype (K1, MAD20 and RO33) of each peptide are indicated down the right hand side of the diagram.

A panel of 132 N-terminal biotinylated 12-mer peptides, representative of all major linear epitopes in the Block 2 region of MSP-1 were used to fine-scale map antibody responses in all immunized and responding animals. This panel included all known repetitive sequences from the K1 and MAD20 serotypes, peptides from all non-repetitive regions of all three serotypes, and a panel of 19 peptides covering the junctions between the individual Block 2 serotypes. Due to the reduced sensitivity of this assay with small volumes of available sera, animals that were seronegative were not tested. In the remaining 25 mice, 6 rabbits and 5 rhesus monkeys, reactivity with peptide epitopes was observed across all three Block 2 serotypes, with some exceptions. Generally, mice showed a more oligoclonal response to Block 2, with a narrower number of reactivities, and with less broad recognition of individual variant peptides (Fig. 6, panel A). Mapping of sera from rabbits showed a broader reactivity, recognising epitopes from all three serotypes (Fig. 6, panel B). Immunized rhesus monkeys showed antibody reactivity with epitopes throughout all three Block 2 serotypes, and had consistently fewer positive reactions with peptides containing putative “neoepitopes” at the junctions between individual Block 2 serotype sequences (Fig. 6, Panel C). In these outbred animals, there was no selective recognition of some epitopes rather than others, which is consistent with our (unpublished) data and that of others for Block 2 antibody reactivity in both animal and human sera [37]. Peptides representing the junctions between the three Block 2 serotypes, which would not occur in natural MSP-1 molecules were included in the 132 peptides tested, to detect any responses directed against potential ‘neo-epitopes’ generated by fusion of the three Block 2 serotypes within a single polypeptide chain. Almost all antibody reactivity in individual serum samples that showed reactivity with the ‘non-natural’ junctional peptides was explained by antibody specificities to peptides from individual ‘natural’ Block 2 sequences.

### Antigenicity in humans

Serum samples from a cross-sectional study conducted in Burkina Faso in 2002 [38] were used to assess the antigenicity of the MSP-1 hybrid in malaria-exposed individuals. Plasma samples from a total of 90 and 55 children, aged <10 years, were collected from Fulani and Mossi ethnic groups respectively. These samples were collected under ethical clearance from the Centre National de Recherche et Formation sur le Paludisme (CNRFP), Ouagadougou. We also included as negative controls 12 serum samples from European blood donors who had not been exposed to malaria.

**Figure 7:**
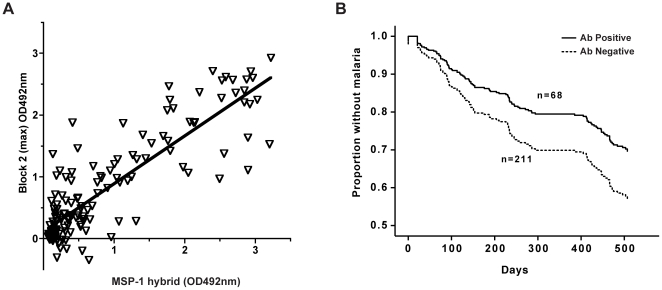
Recognition of MSP-1 hybrid protein by malaria-exposed human sera. A. Reactivity of human sera with MSP-1 hybrid. 145 serum samples from children naturally exposed to malaria in Burkina Faso were tested for reactivity with the MSP-1 hybrid by ELISA. Sera were diluted 1∶500 and tested against the five individual Block 2 GST fusion proteins described in Materials and Methods. For each individual serum sample, antibody reactivity against the MSP-1 hybrid (X axis) is plotted against the maximum Ab reactivity seen against any one of the five individual Block 2 proteins (Y axis). Marginally negative OD values on the Y axis are explained by high reactivity with GST alone in some sera. B. Association between antibodies reactive with the MSP-1 hybrid and reduced incidence of clinical malaria in Ghanaian children over 500 days. Serum samples (diluted 1∶500) from 278 children were tested by ELISA for antibody reactivity with the MSP-1 hybrid. Children were divided into those who were antibody positive (solid line, n = 68) or Ab negative (dashed lined, n = 211) for the MSP-1 hybrid. Survival plots generated by Cox regression analysis of the proportions of children who remained malaria free in the antibody-positive and antibody-negative groups are shown.

As shown in Figure 7A, IgG antibodies from these children specifically recognized the MSP-1 hybrid, and this reactivity correlated with the reactivity in each serum sample against individual Block 2 antigens. Negative control sera did not recognize the protein (data not shown). Of the 145 plasma samples tested, the majority of MSP-1 Block 2 positive sera showed as strong or stronger antibody reactivity with the MSP-1 hybrid protein as was observed with any single serotype MSP-1 Block 2 antigen. This antibody reactivity with the MSP-1 hybrid correlated closely with the maximum reactivity against any single Block 2 antigen (Spearman's Rho = 0.7881, p<0.0001).

Sera from a cohort of Ghanaian children who were part of a seroepidemiological survey of malaria incidence and immune responses [25] were also tested for reactivity with the MSP-1 hybrid. This cohort was clinically and parasitologically monitored for malaria episodes in a period lasting up to 500 days post sampling [25]. It was clear that children with antibodies reactive with the MSP-1 hybrid were significantly less likely to experience a clinical malaria episode during the follow-up period, compared to those who were seronegative for MSP-1 hybrid reactivity (Fig. 7B). Survival analysis using Cox's proportional hazards regression model revealed a clear association between IgG to the MSP-1 hybrid and the time free from malaria (Exp(B) = 0.484, p = 0.006). However, when age was included as a covariate in the analysis, this association was marginally weaker (Exp(B) = 0.642, p = 0.098).

### Parasite growth inhibition assays

**Figure 8:**
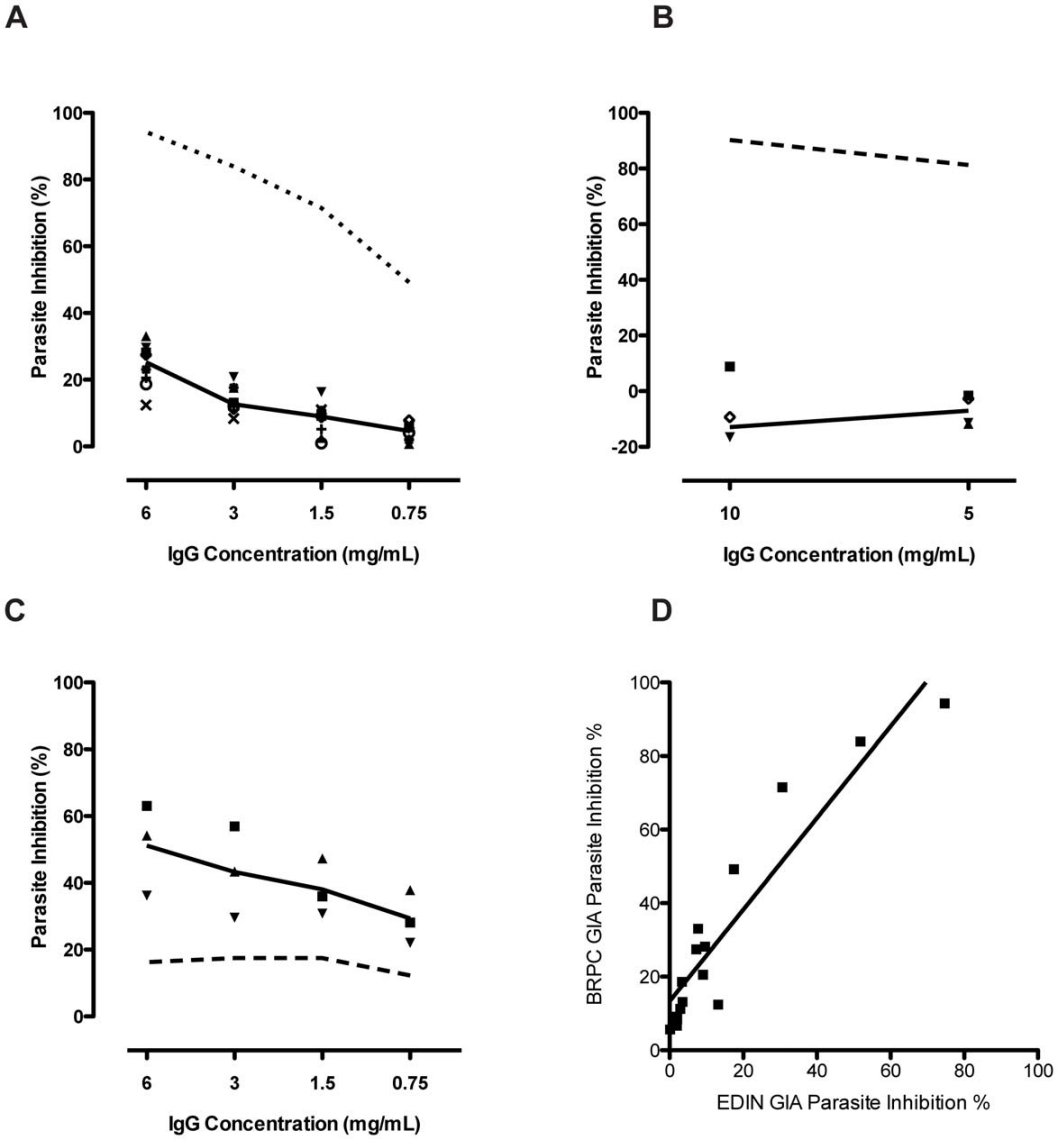
*P. falciparum* growth inhibition assays (GIA) using purified IgG from MSP-1 hybrid immunized animals. Individual symbols show percentage inhibition for IgG from each animal. The solid line indicates the median level of inhibition at each IgG concentration. In panels A and B, the dotted line indicates inhibition obtained with an anti-AMA1 positive control rabbit IgG preparation (BG98 from BPRC Netherlands). A). Growth inhibition using IgG from immunized rabbits and parasite strain 3D7. B) Growth Inhibition using IgG from immunized Rhesus monkeys and strain NF54 C) Growth Inhibition using IgG from an MSP-1 hybrid immunized sheep using parasite strains Wellcome, RO33, and MAD20. Individual symbols represent percentage inhibition of *P falciparum* strains Wellcome, RO33 and MAD20. Solid line; arithmetic mean of these values. Dashed line; growth inhibition using purified naïve sheep IgG. D) Scatterplot comparing growth inhibition assay results from two independent laboratories. Data (from panel A) generated at the University of Edinburgh plotted against data generated at BPRC, Netherlands using identical rabbit IgG preparations. The relationship between the datasets was assessed by linear regression and the line of best fit is plotted as a solid line on the graph (Slope = 1.25, R^2^ = 0.84).

*In vitro* parasite growth inhibition assays were used to assess one functional activity of antibodies against *P. falciparum* parasites. Using purified IgG from final bleed sera, taken from rabbits, sheep and rhesus monkeys, antibodies were tested at a range of concentrations against a number of different parasite strains. Figure 8 shows results from these assays.

Although seven of the eight immunized rabbits responded well to the vaccine, purified IgG from all animals failed to substantially inhibit parasite growth *in vitro*, even at the highest concentration (6 mg mL^−1^) of IgG (median inhibition 25.3%, range 12.4% to 33.0%, Fig. 8, panel A). Similarly, purified IgG from the four immunized rhesus monkeys were also non-inhibitory at both 10 mg mL^−1^ and 5 mg mL^−1^ in GIA (median inhibition −12.9%, range −26.5% to 8.8%, Fig. 8, panel B). This was in contrast to the high levels of inhibition seen in both assays by the control standard rabbit IgG raised against seven different variants of AMA-1 (EMVDA GIA standard, batch No. BG98, from BPRC, Netherlands). By contrast, significant growth inhibition was observed using polyclonal IgG purified from sheep immunized with the MSP-1 hybrid (mean inhibition across three parasite strains 51.2% at 6 mg mL^−1^ IgG, range 36.2% to 63.0%, Fig. 8, panel C). Naïve pre-immunization IgG from the same animal showed little or no growth inhibitory effect, and the low inhibition observed showed no titratable effects (Fig. 8, Panel C, dashed line). It must be noted that IgG from this animal was produced using immunization with Freund's adjuvant, and therefore may have a more inhibitory effect due to higher affinity and/or broader specificity, or may be an artifact of the species of antibody itself, which has not been routinely used for parasite growth inhibition assays. These assays of parasite growth inhibition are reproducible in our hands and in the case of the anti-MSP-1 hybrid rabbit IgG used here (Figure 8, Panel A), there is good correlation between the data produced in our laboratory and that of BPRC, which also tested the same samples (Figure 8, Panel D, R^2^ = 0.844).

## Discussion

This paper reports the expression of a soluble recombinant protein (MSP-1 hybrid) which encompasses the sequence and antigenic diversity present within the majority of MSP-1 Block 2 alleles found within the *P. falciparum* parasite population. A codon-optimised construct was designed to include the known allelic diversity in MSP-1 Block 2, using all polymorphic Block 2 sequences known to be the recognised by human and mouse antibodies elicited by natural infection and immunisation respectively. MSP-1 hybrid contains diverse B cell epitope sequences derived from all three Block 2 serotypes, plus the N-terminal MSP-1 Block 1 sequence known to contain human T cell epitopes [34], [35]. The recombinant protein was expressed with high yield and was purified to homogeneity using a simple three-step process, which was shown to be easily scalable. Promisingly, MSP-1 hybrid yields at fermentor scale and the purity of the final product were comparable to those achieved from shaking flask cultures.

Expression of this hydrophilic, polar domain of MSP-1 in the heterologous host *E.coli* was straightforward, as this region of the MSP-1 gene lacks disulphide bonds and has a low frequency of hydrophobic residues within its amino acid sequence. The production of any construct based on Block 2 sequences is therefore not constrained by disulphide bond formation or the need for complex folding or refolding conditions in production, as Block 2 sequences are intrinsically unstructured protein domains, due to their unusual amino acid content [39], [40]. Codon optimisation of the MSP-1 hybrid construct also enabled even higher-level expression than has been seen with non-optimised Block 2 proteins [41]. Intrinsically unstructured proteins (IUPs) have several unique properties, conferred on them by their unusual amino acid composition, which includes high frequencies of small hydrophilic amino acids. As IUPs have no hydrophobic core, they do not lose solubility at elevated temperatures. Their unusual amino acid composition allows less SDS to bind in SDS-PAGE. This means the apparent Mw of IUPs is often higher than calculated from sequence data or determined by mass spectrometry, and their lack of hydrophobic residues prevents efficient binding by dyes such as Coomassie Blue [40]. The unstructured flexible conformation(s) of the protein also makes fine-scale mapping of a large number of B cell epitopes in these proteins using peptide ELISA possible, which is not always the case for globular proteins that possess more rigid conformational structures.

The thermostable nature of the MSP-1 hybrid also simplified the development of a purification protocol for this vaccine, and gave remarkable stability to the purified product, which remained almost unaltered in liquid form (as estimated by mass spectrometry and SDS-PAGE) over incubation periods of up to 60 days at 25°C and 37°C. This contributes very positively to the suitability of the MSP-1 hybrid as a malaria vaccine component, by removing the requirement for a consistent cold chain.

Purified MSP-1 hybrid showed potent immunogenicity in mice using five different human-compatible adjuvants. Despite its relatively small size (31 kDa), MSP-1 hybrid elicited polyclonal antibodies reactive with *P. falciparum* parasites of all three Block 2 serotypes in mice and rabbits. Furthermore, and very significantly, the vaccine was also immunogenic in pre-clinical testing in non-human primates. Three doses of MSP-1 hybrid formulated in CoVaccine HT elicited potent immunogenicity in Rhesus monkeys. Significantly, sera from animals immunized with the MSP-1 hybrid display a broad reactivity with all three MSP-1 Block 2 serotypes in both IFA, ELISA and peptide mapping.

MSP-1 hybrid was consistently and reliably recognised by African human serum antibodies and importantly by individual human sera that contained single Block 2 specificities, as well as by mouse monoclonal antibodies to Block 2 raised by immunisation with parasite extracts. The antigenic integrity of the MSP-1 hybrid indicates that this vaccine construct contains epitopes recognised by antibodies from naturally exposed individuals and correlates closely with the individual Block 2 reactivities seen in the same serum samples using individual Block 2 antigens. In agreement with previous studies showing that Block 2 epitopes are the target of antibodies associated with protection from clinical malaria [3], [7], [24], [25], in this study antibodies to the MSP-1 hybrid showed a clear association with reduced malaria incidence in a cohort of Ghanaian children. Although the statistical significance of this association was confounded by age, it remained close to significance in a relatively small cohort (278 children) where Block 2 antibody positivity frequency is relatively low and where there was a wide age range (3–15 years).

Antibodies to the MSP-1 hybrid raised by immunization in rabbits and rhesus monkeys showed little growth inhibitory effect *in vitro* in standard GIA. These are not unexpected results, as even high titres of antibodies to several of the merozoite antigens have also been shown to be non-inhibitory in GIA, primarily because these antibodies are against antigens shed or non-covalently associated with the parasite surface (e.g. MSP-2, MSP-3, GLURP) [32], [42], [43], [44]. These antigens however are targets of antibody dependent cellular inhibition (ADCI), a property of such antigens, including MSP-1 Block 2 [30].

Any sustained reduction in host mortality, morbidity and parasite transmission will rely on a vaccine which targets multiple antigens and multiple stages of the parasite lifecycle, thus maximising the “hit” of any putatively effective vaccine. This is clearly in line with the goals of Malaria Vaccine Technology Roadmap, which includes the aim to “pursue multi-antigen, multi-stage, and attenuated whole-parasite vaccine approaches” (www.malariavaccine.org/files/Malaria_Vaccine_TRM_Final_000.pdf -page 4). Thus, development of any blood stage malaria vaccine needs to address the issue of parasite antigenic diversity, as mono-allelic vaccines have been shown to be ineffective [45] or effective against only homologous parasite strains or serotypes [46], [47]. Blood stage malaria parasite antigens are often polymorphic, and this is now being addressed by development of vaccines containing multiple forms of the same parasite antigen, to be delivered as mixtures or as multimeric vaccine constructs [48], [49], [50]. Rather than focus on conserved domains of candidate malaria vaccine antigens, the MSP-1 hybrid addresses the issue of polymorphism directly, by incorporating sequence and antigenic polymorphism into a single protein vaccine product. The recent report that the Block 2 region of MSP-1 is a target of antibody-dependent cellular parasite inhibition activity *in vitro* [30] also supports the further development of a blood stage vaccine based on MSP-1 Block 2. A blood stage (and theoretically liver stage [19], [20]) vaccine based on the MSP-1 hybrid is supported by the evidence presented here, including the biophysical properties of the protein, its strong immunogenicity in all three species of immunized animal, especially in non-human primates, and the ability of the antigen to elicit serotype spanning immune responses recognising multiple parasite strains. A vaccine targeting the extensive antigenic polymorphism of *P. falciparum* could substantially aid in achieving the goals of the Global Malaria Action Plan.

## Materials and Methods

### Ethics statement

All mouse experimentation was carried out in accordance with the Animals (Scientific Procedures) Act 1986 and conforms to the Recommendation from the Declaration of Helsinki and the Guiding Principles in the Care and Use of Animals. The University of Edinburgh Ethical Review Committee approved the project license under which all mouse experimentation was performed on 3rd July 2006, reference number PL 13-06. Mice were humanely killed by Schedule 1 methods.

Rabbit housing and immunization were at BioGenes GmbH (Berlin, Germany), and were in accordance with national and international animal welfare regulations. Rabbit immunization at this facility was under approval from NIH/OLAW (ID number #A5755-01).

Rhesus monkeys used in this study were captive bred for research purposes, and housed at the Biomedical Primate Research Center, Rijswijk (BPRC). Animal care procedures at BPRC are in compliance with Dutch law on animal experiments, European directive 86/609/EEC, and with the “Standard for Humane Care and Use of Laboratory Animals by Foreign Institutions”, identification number A5539-01, provided by the Department of Health and Human Services of the US National Institutes of Health (NIH). All work with Rhesus monkeys is compliant with the recommendations of the Weatherall report “The use of non-human primates in research” (http://www.acmedsci.ac.uk/images/project/nhpdownl.pdf). The independent ethics committee at BPRC, constituted according to Dutch law on animal experiments, approved the study protocol (number DEC598) prior to start of the experiment. Venapunctures and immunizations were performed on ketamine-sedated monkeys to minimise animal stress. Every day animals were carefully observed for their general health. A veterinarian was available at all times to take appropriate action in the event that abnormalities were observed. One animal failed to recover from sedation on day 56 and died due to causes not related to vaccination, as determined by autopsy. During the course of the study all animals received a variety of enrichment items (e.g. toys, food puzzles).

### Design and production of MSP-1 hybrid construct

MSP-1 Block 2 sequences from laboratory strains, from field isolates of *P. falciparum* and downloaded from Genbank were analysed and were all confirmed to belong into three main serotypes, designated K1 type, MAD20 type and RO33 type, after representative parasite clones in each group. Parasites of both the K1 and the MAD20 types have conserved but serotype-specific flanking sequences enclosing a region containing various combinations of tripeptide repeats. Parasites of the RO33 serotype were relatively conserved and contained no repeat sequences, but had some point mutations between isolates within the serotype.

On examination of the aligned protein sequences of the three Block 2 types, plus our data from earlier ELISA-based seroepidemiological surveys of African sera [6], [7], [25], [33], [51], [52], [53], [54], we designed Block 2 sequences containing synthetic tripeptide repeat sequences in an arrangement similar to that found in naturally occurring Block 2 alleles. We designed codon-optimised synthetic versions of both the K1- and MAD20- serotypes of Block 2, both longer than any of the naturally occurring sequences, and containing all of the possible nonapeptide repeat combinations seen within the naturally occurring Block 2 sequences. We also included the type-specific flanking sequences common to each Block 2 type. In addition, we had evidence from previously published work [34], [35] and from our own use of computer-based predictive algorithms, that known and predicted human T-cell epitopes exist within the flanking sequences of Block 2, in the junction region between Block 2 and Block 1, and in Block 1 itself, but are not predicted to exist within the repeats themselves. Since there are no known or predicted T cell epitopes wholly within the Block 2 region itself, and on the basis of earlier immunogenicity testing, we included the Block 1 sequence in the construct to provide cognate T cell help. The final construct consisted of an upstream sequence corresponding to the K1 MSP-1 Block 1 DNA fragment followed by the 3 main variants of the Block 2 derived from Block 2 sequences from K1, RO33 and MAD20 serotypes respectively (Fig. 1). The synthetic gene, called MSP-1 hybrid, encodes a 348 amino acid protein with a predicted molar mass of 31.1 kDa. This gene was synthesised by GeneArt AG, Regensburg, Germany and cloned into the pET24a expression vector (Novagen, UK).

### Flask-based MSP1 hybrid protein expression

The expression plasmid was transformed into chemically competent BL21 (DE3) pLysS *E. coli* (Agilent Technologies Ltd, Stockport, UK) and plated on LB agar plates containing 50 ug mL^−1^ kanamycin. Starter cultures of 10 mL LB broth containing 50 ug mL^−1^ kanamycin and 0.5% w/v L-glucose were inoculated with a single colony from a freshly streaked agar plate. Erlenmeyer flasks containing 1 litre LB broth, 0.5% w/v L-glucose and 50 ug mL^−1^ kanamycin were inoculated with 5 mL starter culture. Culture growth was monitored until the optical density reached 0.6 AU cm^−1^, then protein production was induced by addition of IPTG to 1 mM final concentration. Cultures were incubated for a further 4 hours shaking at 30°C then cells were harvested by centrifugation at 5000× G and 4°C for 15 minutes. Cell pellets were stored frozen at −80°C until further processing.

### Fed batch MSP1 hybrid protein expression

A 15-litre stirred bioreactor (Applikon Technologies, UK) was filled with 8 litres of modified YT medium. pH control was set to maintain pH at 7.2 by addition of 4N H_2_SO_4_ or 30% v/v NH_4_OH. Dissolved oxygen was monitored and maintained at 20% by manually increasing air flow or agitation speed, and temperature was maintained at 37°C. The bioreactor was inoculated by addition of 2% v/v starter culture and culture density was monitored throughout the experiment by measurement of optical density (OD) and wet pellet mass. Upon reaching an OD of 1.2 AU cm^−1^, batch feeding commenced by pumped addition of feed medium at an initial rate of 6.48 mL L^−1^ h^−1^ with an exponential increase in feeding rate (μ = 0.1 h^−1^). Protein expression was induced by addition of 1 mM final concentration of IPTG once a wet pellet weight equivalent to 75 g L^−1^ was achieved. Fermentation continued for a further 4 hours with the continued maintenance of parameters as described above. Cells were harvested by centrifugation at 5000× G and the cell pellet was stored in aliquots at −80°C until further processing.

### Lab Scale Antigen Purification

Cell pellets from flask culture were ruptured by freeze-thaw lysis in 25 mM Tris 250 mM NaCl pH 8.0, treated with 1 U ml^−1^ of DNAse (Benzonase, Novagen), incubated for 20 minutes at 4°C and heated to 70°C for 20 minutes in a heated water bath. The solution was incubated at 4°C for a further 20 minutes and precipitated proteins removed by centrifugation at 5000× G for 30 minutes. The recovered solution was dialyzed extensively against 25 mM Tris buffer pH 8.0, and anion exchange chromatography carried out using a 25 mL Q Sepharose XL column (GE Healthcare, UK). A step gradient of 20 mM NaCl in 25 mM Tris pH 8.0 was used to remove contaminants before elution using 50 mM NaCl in 25 mM Tris pH 8.0. Retained fractions containing the hybrid protein were pooled, dialyzed against PBS and concentrated by centrifugal ultrafiltration using a 10 K cutoff Amicon concentrator (Millipore, UK) before being heat treated at 70°C for storage at −20°C. The final purified protein antigen concentration was measured by BCA assay (Pierce, UK).

### Antigen Purification from fed batch culture

Cell pellets from fed-batch fermentation were resuspended in 10 mL g^−1^ 25 mM Tris pH 8.0 and ruptured by freeze-thaw lysis. The lysate was treated with 2 U ml^−1^ of DNAse (Benzonase, Novagen, UK), incubated for 30 minutes at room temperature then heated to 90°C by passage through a 250 mL stainless steel heat exchanger with a 20 minute residency time. The solution was cooled to 4°C and precipitated proteins were removed by centrifugation at 5000× G for 30 minutes. The recovered solution was dialyzed extensively against 25 mM Tris pH 8.0 buffer, and anion exchange chromatography carried out using a chromatography column containing 200 mL Capto Q bioprocess grade sepharose (GE Healthcare, UK). Column chromatography and further downstream processing were performed as described for the lab-scale purification method.

### SDS-PAGE, Silver staining and Western blot

Bacterial lysates, purification samples and purified MSP-1 hybrid were resolved by sodium dodecyl sulfate-polyacrylamide gel electrophoresis (SDS-PAGE) [55] and gels stained with silver nitrate [56], as MSP-1 hybrid does not stain with Coomassie Blue. The proteins separated by SDS-PAGE were electrophoretically transferred to Schleicher and Schuell BA83 nitrocellulose membranes (Whatman, UK). Membranes were blocked for 1 h using nonfat milk in PBS containing 0.05% Tween 20 (PBS-T), and then probed using antibody mAb 12.2, which specifically recognises the K1-type allele of MSP-1 Block 2 [57]. After washing, horseradish peroxidase-labeled anti-mouse IgG (Dako, UK) was added for 1 h. The blots were washed and developed with 0.6 mg mL^−1^ 4-chloronapthol/0.003% H_2_O_2_. For stability testing, aliquots of purified MSP-1 incubated at 25°C were removed at various time intervals and then stored at −20°C before analysis by SDS-PAGE and silver staining.

### Protein analysis, estimation of purity and stability testing

Samples of the MSP-1 hybrid protein from each step of purification were tested for purity and protein identity using SDS-PAGE, ELISA and Western blotting. ImageJ image analysis software was used to calculate percentage purity of protein samples on SDS-PAGE by densitometry.

For stability testing, samples were heat treated at 70°C for 20 minutes and stored at −70°C, −20°C, 4°C, 25°C and 37°C with sampling for up to 60 days. All time point samples were resolved by SDS-PAGE and stained by silver staining to assess degradation. Molecular weight and pI of the MSP-1 hybrid protein was predicted using the ExPASy ProtParam tool (http://www.expasy.ch/tools/protparam.html) based on its amino acid sequence.

### Mass spectrometry

Mass spectrometric analysis of the purified MSP-1 hybrid protein was carried out by the SIRCAMS (Department of Chemistry, University of Edinburgh, http://www.sircams.ed.ac.uk). For LC-MS, an Ultimate 3000 HPLC system (Dionex Corporation, Sunnyvale, CA), equipped with a monolithic PS-DVB (500 µM×5 mm) analytical column (Dionex Corporation), was used. Samples containing ∼1 µg of protein were centrifuged (16,100× G for 2 min) immediately prior to injection onto the column. Solutions B and C comprised of 2∶97.95 and 80∶19.95 actonitrile∶water with 0.05% formic acid respectively. Samples were injected onto the analytical column, washed with buffer B for 5 min, followed by a 20 min linear gradient elution (20 µL/min) into buffer C. MS data was acquired on a Bruker 12 Tesla Apex Qe FT-ICR (Bruker Daltonics, Billerica, MA) equipped with an electrospray ionization source. Desolvated ions were transmitted to a 6 cm Infinity cell® penning trap. Trapped ions were excited (frequency chirp 48–500 kHz at 100 steps of 25 µs) and detected between m/z 600 and 2000 for 0.5 s to yield a broadband 512 Kword time-domain data. Fast Fourier Transforms and subsequent analyses were performed using DataAnalysis (Bruker Daltonics) software. Multiple charge states could be observed in this way for each of the major species.

### MSP-1 Block 2 production, formulation and immunizations

For immunogenicity studies on a single MSP-1 Block 2 protein derived from the FVO MSP-1 sequence, purified GST Block 2 protein was prepared as previously described [33]. To determine the immunogenicity of the FVO Block 2 protein alone, the FVO Block 2 sequence was cleaved from GST-FVO Block 2 fusion protein by incubation with thrombin at 37°C for 1 hour. FVO Block 2 was purified from residual GST-FVO Block 2 and GST by passage of the proteolytically cleaved material over a GSTrap (Glutathione Sepharose) column five times. The resulting material was then centrifuged through a Vivaspin 6 centrifugal concentrator with 10,000 Da cutoff membranes to remove thrombin (36 kDa) and any potential residual GST (28 kDa). The resulting FVO Block 2 protein was used in immunogenicity studies in comparison with the GST-FVO Block 2 protein from which it was derived.

Outbred MF1 mice or inbred CBA/Ca mice (5 per group, female) were immunized s.c. at 2 sites with either a) MSP-1 FVO Block 2 protein or b) GST-FVO Block 2 formulated with Alhydrogel (Brenntag Biosector, Denmark). For each mouse, 200 µL volumes containing 50 µg protein antigen in 0.32% Alhydrogel were used for each dose. Each dose contained 0.16 mg Al^3+^. Groups of five mice were also immunized with the same doses of each antigen, but formulated by emulsification with Montanide ISA51 adjuvant (ratio 1∶1) (Seppic, France), following the manufacturer's formulation instructions. For all groups of mice, three doses were given at 28 day intervals, the animals were exsanguinated at day 70 and serum prepared from each.

### MSP-1 hybrid antigen formulation and immunizations

Purified sterile MSP-1 hybrid protein antigen was used as the immunogen in the following immunization studies.

1. Outbred female MF1 mice (5 per group) were immunized s.c. with the MSP-1 hybrid formulated with Alhydrogel (Brenntag Biosector, Denmark). For each mouse, 100 µL volumes containing 20 µg protein antigen in a) 0.32% Alhydrogel adjuvant alone or b) in combination with 10 µg/mL of CpG 7909 were used for each dose. Each dose contained 0.16 mg Al^3+^. Groups of five mice were immunized with the same doses of MSP-1 hybrid formulated by emulsification with c) Montanide ISA51 adjuvant (ratio 1∶1) and d) Montanide ISA720 (ratio 7∶3) (Seppic, France), following the manufacturer's formulation instructions. A group of ten mice were immunized with the same dose of MSP-1 hybrid formulated in e) CoVaccine HT™ (Protherics Medicines Development Limited, A BTG International Group Company, London, UK) following the manufacturer's instructions and as previously described [58]. CoVaccine HT contains 40 mg mL^−1^ of Sucrose Fatty Acid Sulphate Esters [SFASE] in a squalane o/w emulsion). A 100 µL vaccine dose contained 20 µg of antigen and 2 mg of SFASE. For all groups of mice, three doses were given at 28 day intervals contemporaneously, the animals were exsanguinated at day 70 and serum prepared from each.
2. Eight New Zealand White rabbits were immunized intramuscularly, using 50 µg per dose of MSP-1 Hybrid antigen formulated with CoVaccine HT following the manufacturer's instructions. A 500 µL vaccine dose contained 50 µg of antigen and 10 mg of SFASE. Each animal was immunized 3 times at 28 day intervals, with blood samples taken at days 0, 28, 56 and 70. Rabbit housing and immunization were at BioGenes GmbH (Berlin, Germany), and were in accordance with national and international animal welfare regulations. Rabbit immunization at this facility was under approval from NIH/OLAW (ID number #A5755-01).
3. Five Rhesus macaques were immunized by BPRC, Rijswijk, Netherlands, using 50 µg per dose of MSP-1 Hybrid antigen formulated with Covaccine HT following the manufacturer's instructions and as previously described for formulation of *P. falciparum* apical membrane antigen 1 (AMA-1) [58]. Each animal was immunized 3 times with 0.5 mL volumes containing 10 mg SFASE at 28-day intervals in alternating legs (left, right, left), with blood samples taken at days 0, 28, 56 and 70.

### Monoclonal and polyclonal antibodies

Mouse monoclonal and polyclonal antibodies for which specific MSP-1 Block 2 epitopes had been confirmed in earlier studies [33], [59] were used for characterisation of the MSP-1 hybrid by ELISA and Western blotting. Sera from 145 children from Burkina Faso who lived in an area of seasonal malaria incidence [38] were used to test the antigenicity of the MSP-1 hybrid by ELISA. Samples of 278 Ghanaian children whose clinical malaria status was known were originally collected for seroepidemiological studies [25], [26], [60]. Control sera of malaria-naive Europeans were taken from donors at the Scottish Blood Transfusion Service.

### Antibody Purification

Immunoglobulin G from immunized animal sera was purified on an Äkta Prime™ chromatography system using HiTrap protein G columns, according to the manufacturer's recommended protocols (GE Healthcare). Control IgG antibodies were purified from naïve animals, or from day 0 (pre-immunization) animals for Rhesus monkeys.

### Parasite culture and growth inhibition assays

*In vitro* parasite growth Inhibition assays (GIA) were carried out on freshly thawed clones of *P. falciparum* Wellcome or FCR3 strains according to standard protocols [61], [62], using purified IgG from MSP-1 hybrid-immunized animals. Total IgG purified from a naïve non-immunized animals were used as negative controls, or from day 0 (pre-immunization) serum samples in the case of Rhesus macaques. Rabbit IgG GIA was performed independently at both BPRC and at the University of Edinburgh.

All IgG samples were tested in triplicate at 2-fold serial dilutions, from 6 mg mL^−1^ (rabbit, sheep) or from 10 mg mL^−1^ (Rhesus) in 96-well cell culture plates (Greiner BioOne, UK). Parasites were cultured under standard conditions [63]. Parasite cultures were mycoplasma-free and synchronized at least twice with 5% sorbitol before use in assays. Late trophozoite/early schizont stages at a parasitaemia of 0.3±0.1% and 2% final haematocrit were used in all assays. The final culture volume was 100 µL/well and parasites were incubated for 42 h. After 42 hours, cultures were tested for growth inhibition using pLDH measurement, flow cytometry and Giemsa-stained slide microscopy. The data is presented as the arithmetic mean percentage inhibition from each tested sample.

### Antigens and Enzyme-Linked Immunosorbent Assay (ELISA)

Individual MSP-1 recombinant proteins based on the 3D7, Palo Alto, MAD20, Wellcome and RO33 MSP-1 Block 2 serotypes have been described elsewhere [25], [33]. These GST fusion proteins and purified MSP-1 hybrid were used for ELISA analysis of Block 2 serotype reactivity.

Human sera, monoclonal antibodies and sera from MSP-1 hybrid immunized animals were tested by previously described ELISA for recognition of the MSP-1 hybrid and Block 2 GST fusion proteins [33] [51]. Negative control wells in ELISA were either coating buffer alone for the MSP-1 hybrid, or GST-coated wells for Block 2 GST fusion proteins. All sera were tested across a range of doubling dilutions (1∶1000 to 1∶128,000) against each antigen in duplicate wells, with a standard pool of Block 2 positive sera also tested on the each plate, and across the same dilution range. ELISA titers (arbitrary units) were calculated by interpolation from the fitted standard curve on each plate using polynomial logistic regression.

### ELISA with biotinylated peptides

A set of 133 biotinylated dodecapeptides covering all possible linear epitopes contained within MSP-1 hybrid sequence were synthesised by Mimotopes Pyt. Ltd. (Clayton, Australia). ELISA plates (Immulon 4 HBX, Thermo Dynex) were coated with 100 µL of 5 µg ml^−1^ streptavidin (Sigma) and incubated at 37°C until dry. Plates were stored in heat sealed foil pouches with 1 g silica gel at room temperature until use. Reactivity of sera against the peptide library was determined by ELISA. Streptavidin-coated plates were washed in PBS-T (PBS/0.05% Tween20) and blocked with blocking buffer (1% ByCoA, Croda Healthcare, UK dissolved in PBS) for 5 hours at room temperature. Peptide library plates were prepared by addition of 300 ng peptide per well, in duplicate, and plates were incubated overnight at 4°C. Sera were added to each well (100 uL at 1∶500 dilution) and incubated overnight at 4°C, then washed with PBS-T. Dilutions of a species-specific HRP-linked secondary antibody (Dako, UK), appropriate to the serum being tested, were added to each well and plates were incubated at room temperature for 3 hours. Plates were washed three times with PBS-T and OPD substrate was added to each well. Reactions were stopped by addition of sulfuric acid and absorbance was read at 492 nm using a microplate absorbance reader (Multiskan Ascent, Thermo Scientific, UK). Background reactivity was calculated as the mean of all OD values in the lowest two quartiles (i.e. below median)+6 standard deviations. Peptide reactivity data was then categorized into the following groups; high = background +1 OD, medium = background +0.5 OD, low = greater than background, negative = below background.

### Indirect immunofluorescence assays (IFA)

Serum samples from mice immunized with MSP-1 Block 2 were analyzed by IFA for parasite reactivity with the Wellcome isolate of *P. falciparum* by methods previously described [33]. Wellcome strain was chosen for IFA as it has an identical Block 2 sequence to the FVO isolate. Endpoint titers were calculated as the highest dilution at which clear antibody reactivity with schizont stage parasites could be observed under FITC fluorescence.

### Statistical analysis

For comparisons between ELISA titres in groups of immunized animals, the data were analysed using the non-parametric Kruskal-wallis test, with Dunn's post-test. For comparisons between two immunization groups, the Mann-Whitney U test was used. For correlation plots of human antibody reactivity to Block 2 versus the MSP-1 hybrid, Spearman's rank correlation coefficient was used. Survival analysis of the time to clinical malaria in Ghanaian children was performed by Cox's regression analysis. In all cases, p values<0.05 were considered statistically significant. Plots and analysis were prepared with either the Prism graphical and statistics package (GraphPad Software, Inc., La Jolla, USA), SPSS version 18 (SPSS Inc, Chicago, USA) or the R statistical language (http://www.R-project.org).

## Supporting Information

Figure S1 **IFA titres of sera from mice immunized with either GST-FVO Block2 fusion protein or FVO Block 2 alone.** Sera were tested by IFA against the Wellcome *P. falciparum* strain (identical Block 2 sequence to FVO). Hollow symbols, CBA mice; filled symbols, MF-1 mice. Circles, GST-FVO Block 2 immunized mice; squares, FVO block 2 immunized mice. Horizontal bars mark the median IFA titre for each group.
(TIF)
