# Supplementary figures and images for: A Malaria Vaccine Based on the Polymorphic Block 2 Region of MSP-1 that Elicits a Broad Serotype-Spanning Immune Response
Source: PLoS One. 2011 Oct 26;6(10):e26616. doi: 10.1371/journal.pone.0026616 (PMC3202563; doi:10.1371/journal.pone.0026616)

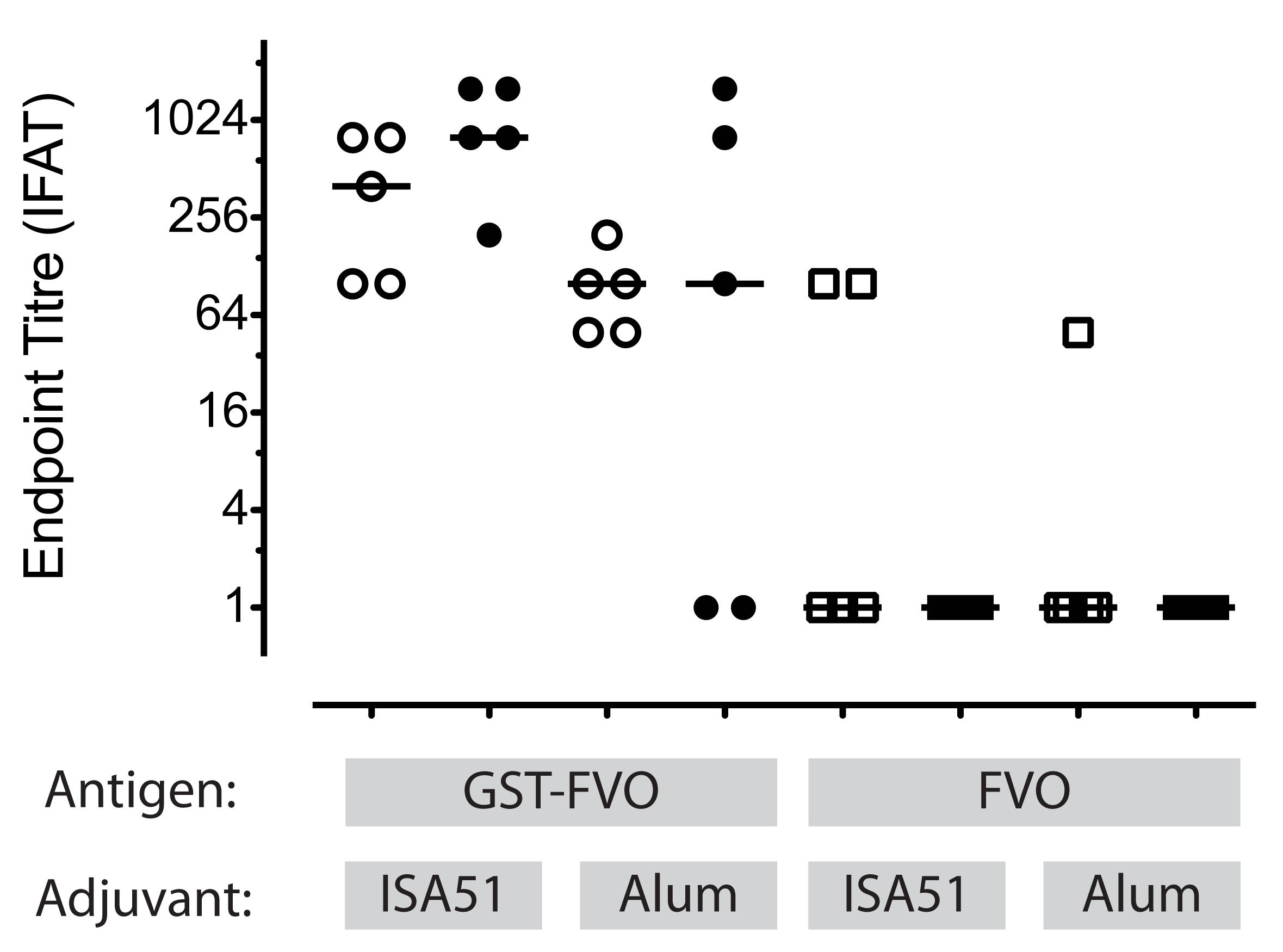

Supplement: Figure S1 — IFA titres of sera from mice immunized with either GST-FVO Block2 fusion protein or FVO Block 2 alone. Sera were tested by IFA against the Wellcome P. falciparum strain (identical Block 2 sequence to FVO). Hollow symbols, CBA mice; filled symbols, MF-1 mice. Circles, GST-FVO Block 2 immunized mice; squares, FVO block 2 immunized mice. Horizontal bars mark the median IFA titre for each group. (TIF) [file pone.0026616.s001.tif]
